# Agnostic evaluation of ipilimumab and nivolumab association: a metanalysis

**DOI:** 10.1186/s12967-020-02588-2

**Published:** 2020-11-25

**Authors:** Paolo Marchetti, Andrea Botticelli, Antonio Paolo Ascierto, Giuseppe Curigliano, Diana Giannarelli

**Affiliations:** 1 Department of Clinical and Molecular Medicine, Sapienza, University of Rome, Policlinico Umberto I, Sant’Andrea Hospital, IDI IRCSS, Rome, Italy; 2 Instituto Nazionale Tumori Istituto di Ricovero e Cura a Carattere Scientifico Fondazione Pascale, Naples, Italy; 3 Department of Oncology and Hemato-Oncology, University of Milano and European Institute of Oncology, IRCCS, Milano, Italy; 4 Biostatistics Unit, National Cancer Institute Regina Elena IRCCS, Rome, Italy

**Keywords:** Immunotherapy, Combination immunotherapy, Nivolumab, Ipilimumab, Agnostic approval

## Abstract

**Background:**

Ipilimumab and Nivolumab, targeting the molecules CTLA-4, PD-1, respectively,have shown efficacy against several types of cancer. Despite these results, only a small percentage of patients maintains a long-lasting effect. Even Ipilimumab, in combination with nivolumab, has demonstrated a significant clinical benefit in multiple tumor types. However, no trial has been designed with the primary endpoint to compare the efficacy of nivolumab plus ipilimumab combined, compared to nivolumab alone. Hence, the added value of ipilimumab in the combination has not clearly been established yet. The aim of this study was to demonstrate the superiority of the combination strategy compared to the single agent therapy.

**Materials and methods:**

We performed a meta-analysis of Phase I-II-III Clinical Trials, published from 2010 up to 2020, in which the combination of ipilimumab plus nivolumab was compared to nivolumab alone. We extracted ORR, OS and PFS HR on the basis of treatment from the subgroup analysis of each trial.

**Results:**

A total of 7 trials were included in the present meta-analysis. Overall, 1313 patients were treated with the nivolumab plus ipilimumab combination compared to 1110 patients treated with nivolumabalone. All trials reported the Objective response rate(ORR), no heterogeneity was found among studies and the pooled Odds Ratio was highly in favor of the nivolumab plus ipilimumab combination with respect to nivolumab alone (1.683; 95% CI: 1.407–2.012; *P* < 0.0001). Three studies were considered for Progression free survival (PFS) analysis, and the pooled Hazard Ratio favored the combination of nivolumab plus ipilimumab with respect to nivolumab alone (0.807; 95% CI: 0.719–0.907; *P* < 0.0001). The Overall survival(OS) endpoint was considered only in 2 trials, and the pooled HR favored, also in this case, the combination of nivolumab plus ipilimumab with respect to nivolumab alone (0.87; 95% CI: 0.763–0.997; *P* = 0.045).

**Conclusions:**

The combination of ipilimumab plus nivolumab seems to be superior to nivolumab alone in cancer patients, regardless of histology.

## Background

Tumour-agnostic therapies target specific gene mutations or molecular features regardless of tumour site of origin [1]. By integrating this definition, if we consider the immune system as the selective target of immunotherapy, an agnostic evaluation (i.e., transversal between the different cancer types) can be made between the associations of two different immunotherapies with respect to the results obtained with only one of these.

Cancer immunotherapies that target the immunosuppressive checkpoint receptors cytotoxic T-lymphocyte-associated protein 4 (CTLA-4) or programmed death 1 (PD-1) and its ligand, programmed death 1 ligand (PD-L1), have changed the landscape of cancer treatment [2]. Ipilimumab therapy first showed a survival advantage in melanoma patients, when compared to a gp100 vaccine or chemotherapy [3]. Nivolumab, targeting PD-1, prolonged overall survival in multiple tumor types including melanoma, non-small cell lung cancer (NSCLC), renal cell carcinoma (RCC), head and neck carcinoma and Hodgkin’s lymphoma. Despite this unprecedented efficacy, many patients fail to respond, presenting primary resistance, and more concerning, some patients who demonstrate encouraging initial responses to immunotherapy, can acquire resistance over time. It has been proposed that mechanisms promoting either primary or acquired resistance are largely conserved, and that they must affect either tumor immunogenicity, antigen presentation and generation of effector T-cells, the encounter of antigen and PD-L1 by tumor-specific T-cells, the activity and efficacy of tumor-specific immune responses or the induction of immunological memory [4]. Considering the elucidated mechanisms of resistance to anti-PD-1, it is reasonable to believe that a more accurate selection of patients and a combination of therapies might yield a greater benefit by enhancing anti-tumor activity. Indeed, Ipilimumab in combination with nivolumab has demonstrated significant clinical benefit in multiple tumor types. From an immunological point of view, it is still unclear whether the enhanced efficacy of the combination of anti–PD-1 and anti-CTLA-4 therapy is mediated by an additive effect of the cellular and molecular mechanisms of the respective therapies or, alternatively, through different and distinct mechanisms of each therapy alone [4]. However, until now, no trial has been designed with the primary end point being the comparison of the efficacy of nivolumab plus ipilimumab versus nivolumab alone and the added value of ipilimumab in the combination.The aim of this analysis is to demonstrate that the addition of ipilimumab to nivolumab results in improved efficacy among multiple solid tumors.

## Patients and methods

### Literature search and inclusion criteria

We identified all randomized trials evaluating the combination of ipilimumab plus nivolumab in different tumor types. Published studies were searched in MEDLINE, EMBASE, BIOSIS and DRUGU and abstracts were looked-up in ASCO and ESMO archives, independently. The following search terms were used: combination immunotherapy, checkpoint inhibitors combination, ipilimumab AND nivolumab.

In all the studies included in the analysis (Table 1), the Objective Response Rate (ORR) was reported; some of these also reported risk reduction (HR) in Progression Free Survival (PFS) and Overall Survival (OS).

**Table 1:** List of clinical trials included in the analysis

Study	Phase	Histology	Masking	No. patients	Treatment arms
CA209-067 [8]	3	Melanoma	Double-blind	945	Nivolumab + Ipilimumab vs Nivolumab vs Ipilimumab*
CA209-227 [10]	3	Nonsmall cell lung cancer (NSCLC)	Open-label	1189	Nivolumab + Ipilimumab vs chemotherapy vs Nivolumab*
IFCT-1501 MAPS2 [11]	2	Mesothelioma	Open-label	108	Nivolumab + Ipilimumab vs nivolumab
Alliance A091401 [12]	2	Sarcoma	Open-label	85	Nivolumab + Ipilimumab vs nivolumab*
CA209-032 [13]	1/2	Small cell lung cancer (SCLC)	Open-label	196	Nivolumab + Ipilimumab and Nivolumab**
CA209-032 [14]	1/2	Gastric	Open-label	108	Nivolumab + Ipilimumab and Nivolumab**
CA209-032 [15]	1/2	Bladder	Open-label	196	Nivolumab + Ipilimumab vs nivolumab

*Nivo 3 mg + IPI 1 mg. **Nivo 1 mg + IPI 3 mg

In studies with multiple treatment arms, we only considered those including patients treated with either nivolumab alone or in combination with ipilimumab.

### Data extraction

Abstract evaluation and data extraction were performed by two reviewers, independently (S.M. and D.G.). In the cases of disagreement, a third reviewer provided support. When the data for the same trial was reported in different papers, the manuscript with the longer patient follow-up was included in this meta-analysis.

Response rate was never the primary endpoint of these studies and ORR was calculated deriving data from the published paper.

### Statistical analysis

Odds ratios (ORs) and their 95% CIs were calculated for ORR as dichotomous outcomes. Hazard ratios (HRs) were summarized, and their corresponding standard errors were derived to analyze PFS and OS. The inverse variance algorithm and the Mantel‐Haenszel algorithm were used. The presence/absence of heterogeneity was evaluated by calculating the Q statistic, a correspondent *P* < 0.05 indicated presence of heterogeneity between studies. A fixed‐effect model and a random‐effect model were used according to the significance of the Q test.

Comprehensive Meta-Analysis software was used for the analysis.

## Results

**Fig. 1:**
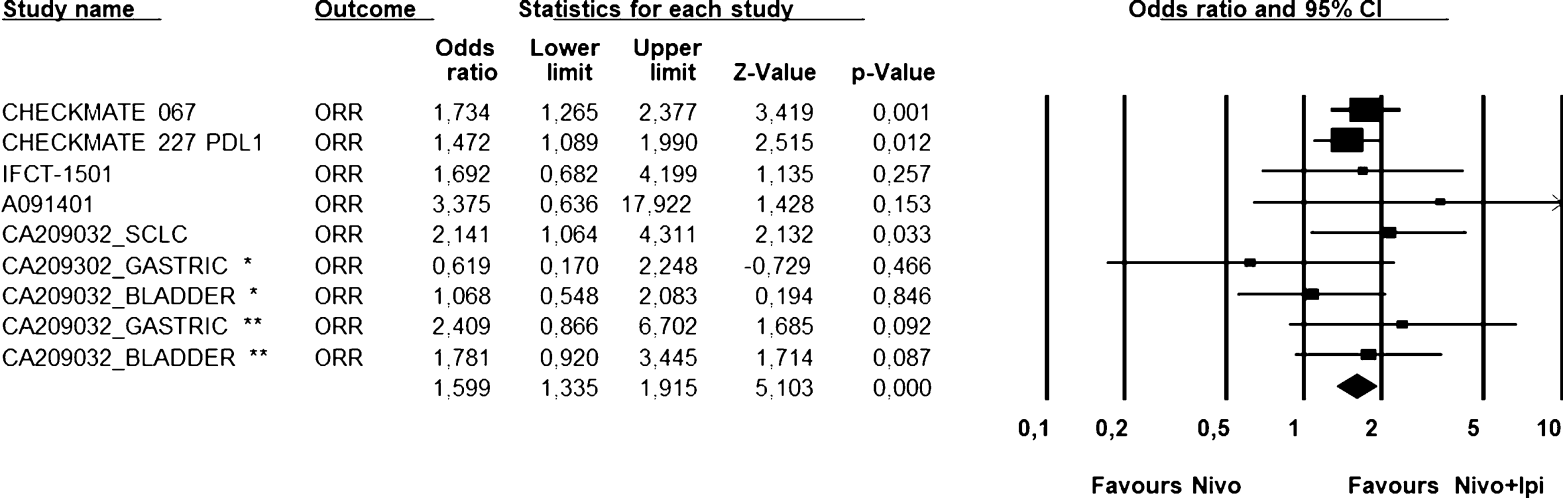
Global ORR Analysis. Figure shows ORR analysis in selected trials. ORR was highly favoring the combination of nivolumab plus ipilimumab with respect to nivolumab alone (OR = 1.683; 95% CI: 1.407–2.012; *P* < 0.0001). Fixed effect model—Heterogeneity not significant (P = 0.62). *Nivo 3 mg + IPI 1 mg. **Nivo 1 mg + IPI 3 mg

**Fig. 2:**
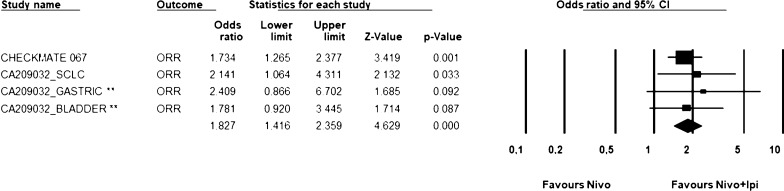
ORR analysis for NIVO1/IPI3. Figure shows the ORR analysis in selected trials in which NIVO1/IPI3 schedule was administered. Fixed effect model—Heterogeneity not significant (*P *= 0.89). NIVO1/IPI3: nivolumab 1 mg/kg and ipilimumab 3 mg/kg

**Fig. 3:**
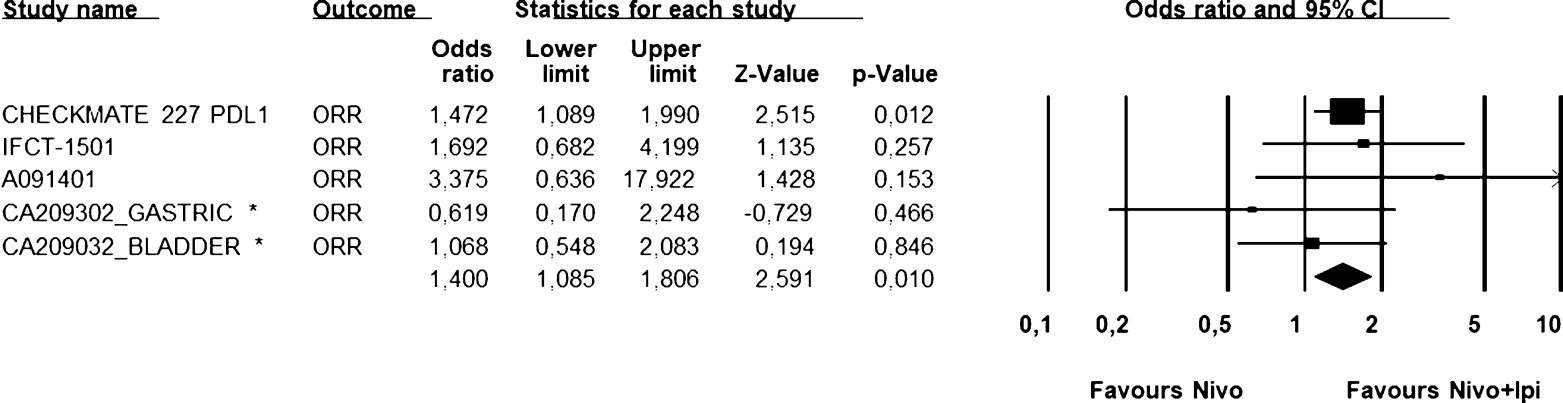
ORR analysis for NIVO3/IPI1. Figure shows ORR analysis for selected trial in which was administered NIVO3/IPI1 schedule. Fixed effect model—Heterogeneity not significant (*P* = 0.48). NIVO3/IPI1: nivolumab 3 mg/kg and ipilimumab 1 mg/kg

**Fig. 4:**
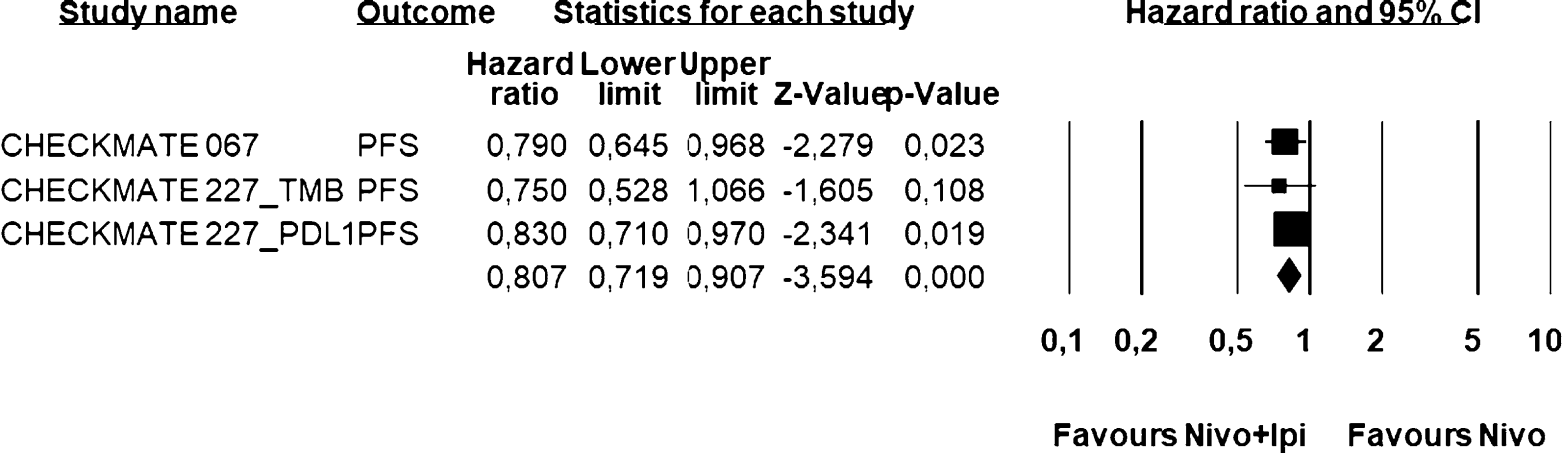
PFS Analysis. Figure shows the PFS analysis on selected trials. Pooled Hazard Ratio favored the combination of nivolumab plus ipilimumab with respect to nivolumab alone (HR = 0.807; 95% CI: 0.719–0.907; *P* < 0.0001). Fixed effect model—Heterogeneity not significant (*P* = 0.85)

**Fig. 5:**
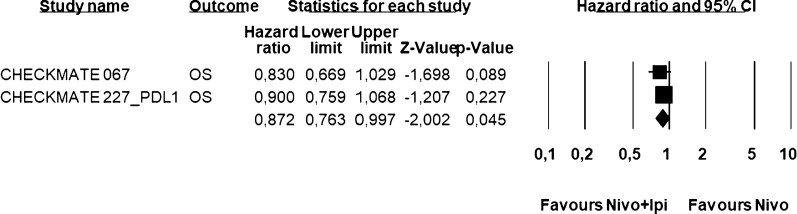
OS Analysis. Figure shows the OS analysis on selected trials. The pooled HR favored the combination of nivolumab plus ipilimumab with respect to nivolumab alone (HR = 0.87; 95% CI: 0.763–0.997; *P* = 0.045). Fixed effect model—Heterogeneity not significant (*P* = 0.56)

A total of 7 trials were included in the analysis; treatment phase, tumor types and treatment arms are reported in Table 1. Overall, this meta-analysis includes 1313 patients treated with the nivolumab plus ipilimumab combination and 1110 patients treated with nivolumab alone. All trials reported ORR (Table 2), the Q statistic (P = 0.94) suggested absence of heterogeneity among studies and the pooled Odds Ratio, based on the fixed-effect model, (Fig. 1) was highly favoring the combination of nivolumab plus ipilimumab with respect to nivolumab alone (OR = 1.683; 95% CI: 1.407–2.012; P < 0.0001). The superiority of combination compared to monotherapy is independent from the schedule of treatment, in particular in 4 studies the schedule was NIVO1/IPI 3 (Fig. 2) while in 5 studies was NIVO3/IPI 1 (Fig. 3). Three studies were considered for PFS analysis (Table 3), also here too, the Q statistic showed a P value equal to 0.85 and the pooled Hazard Ratio (Fig. 4) favored the combination of nivolumab plus ipilimumab with respect to nivolumab alone (HR = 0.807; 95% CI: 0.719–0.907; *P* < 0.0001). The OS endpoint was considered only for 2 trials (Table 4) for which results were in the same direction and the pooled HR (Fig. 5) also favored the combination of nivolumab plus ipilimumab with respect to nivolumab alone (HR = 0.87; 95% CI: 0.763–0.997; *P* = 0.045).

**Table 2:** List of clinical trials included in the ORR analysis

Study	No. patients	ORR
CA209-067	n + i = 314 n = 316	n + i = 58.3% n = 44.6%
CA209-227	n + i = 396 n = 396	n + i = 35.9% n = 27.5%
IFCT-1501 MAPS2	n + i = 54 n = 54	n + i = 27.8% n = 18.5%
Alliance A091401	n + i = 38 n = 38	n + i = 15.8% n = 5.3%
CA209-032 (GastricCancer)	n + i = 49* n + i = 52** n = 59	n + i = 24.5% n + i = 7.7% n = 11.9%
CA209-032 (BladderCancer)	n + i = 196 n = 78	n + i = 34% n = 24%
CA209-032 (SCLC)	n + i = 147 n = 95	n + i = 21% n = 12%

*Nivo 3 mg + IPI 1 mg. **Nivo 1 mg + IPI 3 mg

**Table 3:** List of clinical trials included for PFS analysis

Study	No. patients	Median PFS (months)	HR
CA209-067	n + i = 314 n = 316	n + i = 11.5 n = 6.93	HR 0.79
CA209-227 (PFS TMB High)	n + i = 101 n = 102	1 yr n + i = 42% n = 29%	HR 0.75
CA209-227 (PFS PD-L1 > 1%)	n + i = 396 n = 396	n + i = 5.1 n = 4.2	HR 0.83

**Table 4:** List of clinical trials included for OS analysis

Study	No. patients	Median OS (months)	HR
CA209-067 [1, 2]	n + i = 314 n = 316	n + i = NR n = 36.93	HR 0.83
CA209-227 [4] (OS PD-L1 > 1%)	n + i = 396 n = 396	n + i = 17.1 n = 15.7	HR 0.79

**Fig. 6:**
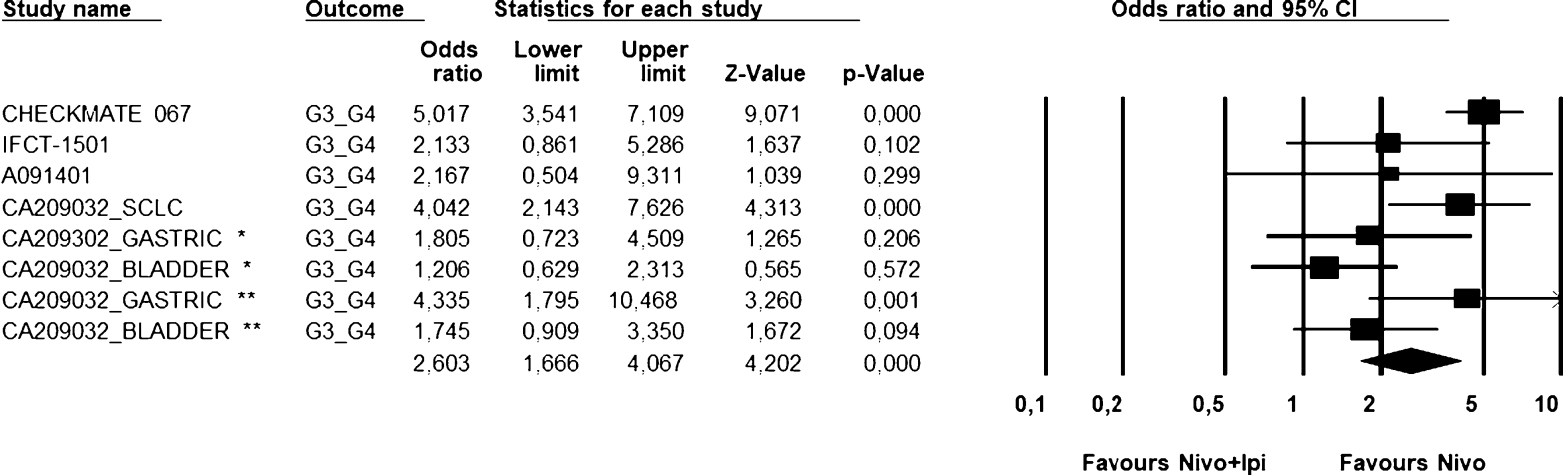
Global G3/G4 toxicity analysis. Figure shows the G3/G4 toxicity analysis for all the selected trials. Nivolumab monotherapy was favored compared to the combination. Random effect model—Significant heterogeneity (*P* = 0.003). *Nivolumab 3 mg + ipilimumab 1 mg **Nivolumab 1 mg + ipilimumab 3 mg

The combination resulted in higher incidence of G3-G4 toxicities as shown in Fig. 6, Additional file 1: Figures S1 and S2.

## Discussion

The number of cancer patients who benefit from immunotherapy has increased due to a better understanding of the immune response to cancer along with recent advances in biomarker development. In particular, an interesting component of immunotherapy is the long-lasting tumor responses observed, with some patients achieving disease control for many years. Nevertheless, not all patients benefit from immunotherapy, and efforts should focus on improving the efficacy of immunotherapy through the use of both combination or sequential approaches and predictive biomarkers of response and resistance [16]. The goal of combination approaches, targeting several steps of the cancer-immunity cycle, is to expand the spectrum of patients who could respond to cancer immunotherapy (increased number of responding patients in tumors that are sensitive to single agent therapy and the identification of new sensitive tumor types that do not respond to monotherapy alone) and to improve the quality of clinical responses (i.e., time span of response, PFS and OS) beyond what can be achieved with monotherapy alone [17]. The aim of such antitumor strategies will be to raise the tail on the survival curve by increasing the number of long term survivors, while managing any additive or synergistic toxicities that may arise with immunotherapy combination. In our analysis, we found that combination therapy was superior to monotherapy. This may have several explanations: (1) the efficacy of monotherapy is limited by low response rates, with only a small proportion of patients responding to treatment; (2) combining anti-CTLA-4 and anti-PD-1 therapies may activate the antitumor immune response synergistically, thus increasing response rates; (3) combining anti-CTLA-4 and anti-PD-1 therapies significantly increases the ratios of both CD8 + /regulatory T cells and CD4 + effector/regulatory T cells within the tumor, so that CD8 + and CD4 + T cells continue to survive, proliferate and carry out effector functions in the tumor; (4) combining anti-CTLA-4 and anti-PD-1 therapies induces the accumulation of active T cells that express CTLA-4 and PD-1 and would otherwise be energized; and (5) combining anti-CTLA-4 and anti-PD-1 therapies increases the production of inflammatory cytokines (such as IFN-γ and TNF-α) in the tumor itself and in its draining lymph nodes.

The scientific rationale of the combination is linked to the evidence that each immunotherapy checkpoint blockade leads to a distinct and non-overlapping signature of changes in T cells and the immune compartment. In particular, several investigators have demonstrated that PD-1 blockade mainly leads to changes in genes implicated in cytolysis and NK cell function, differently from CTLA-4 blockade that induces a proliferative signature in a subset of memory T cells. This activity of ipilimumab on the memory cell compartment may be responsible for the prolonged responses observed in patients treated with this drug. Indeed, although objective antitumor response rates were low (~ 10%), approximately 20% of patients had a long-lasting response up to 10 years and this sustained benefit may represent the potential of anti-CTLA4 immunotherapy in raising the tail of the survival curve. This effect on immunologic memory can be further demonstrated by the observation that less than 4 doses of ipilimumab can be sufficient to induce the long-term effect on the survival curve.

In intermediate/poor risk metastatic renal cell carcinoma, first-line therapy withthe combination ipilimumab plus nivolumab showed that 60% of patients were alive at 30 months, with a 42% ORR and 11% CR. In untreated advanced melanoma nivolumab plus ipilimumab versus nivolumabalone results in higher 5-yr OS [(52% versus 46%) with HR of 0.83 (95% CI, 0.67–1.03)], and PFS [37% versus, 31% with HR 0.79 (95% CI, 0.64–0.96)]. These differences were consistent across many clinically relevant subgroups, including BRAF-mutant patients and poor prognostic subgroups, such as patients with elevated LDH levels and M1c disease. First-line therapy for non-small-cell lung cancer using combination nivolumab plus ipilimumab in patients with a PD-L1 expression level of 1% or more, shows a median overall survival of 17.1 months and 15.7 months with nivolumab alone [HR 0.90 (0.76–1.07)] and a 2 year overall survival rate of 40.0 and 36%, respectively. The median duration of response was 23.2 months with nivolumab plus ipilimumab and 15.5 months with nivolumab. Overall survival benefit was also observed in patients with a PD-L1 expression level of less than 1%, with a median duration of 17.2 months (95% CI, 12.8–22.0) with nivolumab plus ipilimumab. Preliminary but very encouraging results derive from the combination of ipilimumab plus nivolumab in melanoma patients treated in the neoadjuvant setting, achieving 78% of pathological response [18].

In this analysis, we confirmed, in a larger population with several cancer subtypes, the results of Yang and colleagues [19]. In particular we demonstrate that the addition of ipilimumab to nivolumab increases ORR to approximately 68% (range 8–95) and reduces the risk of progression and death of about 20% (range 10–28) and 13% (range 1–24), respectively, regardless of tumor type.

Additional evidences of the improved outcome by adding ipilimumab to nivolumab (“boost” cycles) in metastatic RCC patients, with early significant progressive disease (PD) at week 8 or stable disease (SD) or PD at week 16 during nivolumab induction, has been reported in the Titan trial [20]. Of the 207 patients enrolled in the study, 64.3% (133/207) received at least one “boost” cycle. Overall 29.8% (14/47) of RCC patients in first line treatment and 38.6% (22/57) of patients in second line treatment with SD/PD after nivolumab monotherapy had improvement in best overall response (BOR) with the “boost” cycles, respectively.

From a safety point of view, no new signals have been observed with the combination compared to monotherapy and, despite the higher level of immune relate adverse events (irAE) observed with the combination therapy, it is worth noting that: (1) patients who were required to come off treatment due to irAE had an overall benefit when compared to the entire population and (2) toxicity of the combination appears to be as manageable as single agent immunotherapy and it has been demonstrated that the need to treat irAE with corticosteroids does not impact on outcome.

However, the added benefit of each additional drug must be properly evaluated against the added toxicities, even if no new signals have been observed with the combination compared to the monotherapy.

## Conclusion

The “agnostic evaluation” of the ipilimumab plus nivolumab combination suggests the “agnostic efficacy” of the combination, compared to mono-immunotherapy, in the population selected for immunotherapy treatment, regardless of tumor type.

## Supplementary information

**Additional file 1:** **Figure S1.** G3/G4 toxicity analysis for NIVO1+IPI3 schedule. **Figure S2.** G3/G4 toxicity analysis for NIVO3+IPI1 schedule.

## Data Availability

The datasets used and/or analyzed during the current study are available from the corresponding author on reasonable request.
