# Supplementary material for: Agnostic evaluation of ipilimumab and nivolumab association: a metanalysis
Source: J Transl Med. 2020 Nov 25;18:446. doi: 10.1186/s12967-020-02588-2 (PMC7688006; doi:10.1186/s12967-020-02588-2)
Supplement: Supplementary file 1 — Additional file 1: Figure S1. G3/G4 toxicity analysis for NIVO1+IPI3 schedule. Figure S2. G3/G4 toxicity analysis for NIVO3+IPI1 schedule. [file 12967_2020_2588_MOESM1_ESM.docx]

Figure S1. *G3/G4 toxicity analysis for NIVO1+IPI3 schedule*


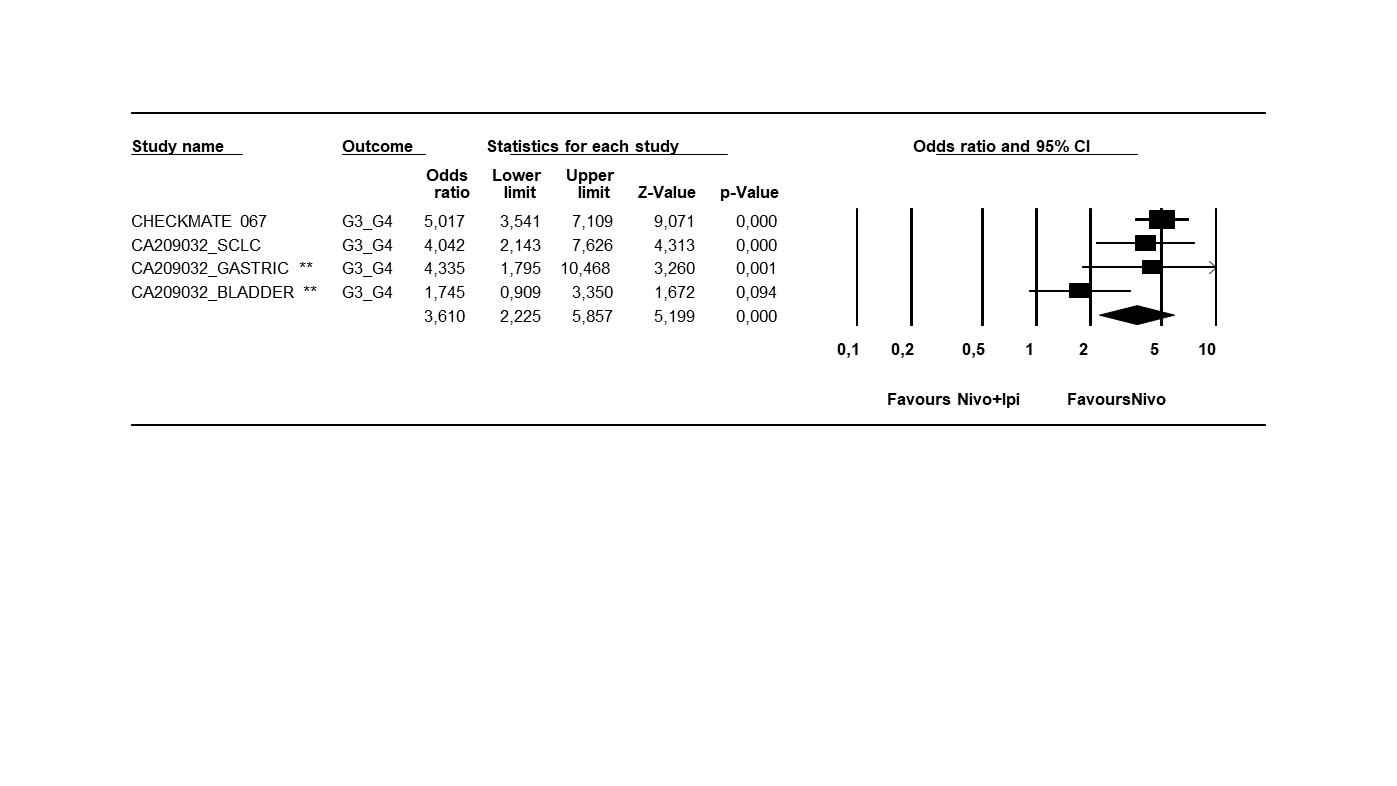


Figure S1 shows G3/G4 toxicity analysis for *NIVO1+IPI3 schedule. Nivolumab had a large benefit compared to NIVO1+IPI3 combination. Random effect model –Significant heterogeneity(P=0.049). NIVO1/IPI3: nivolumab 1 mg/kg and ipilimumab 3 mg/kg.*

*Figure S2. G3/G4 toxicity analysis for NIVO3+IPI1 schedule*


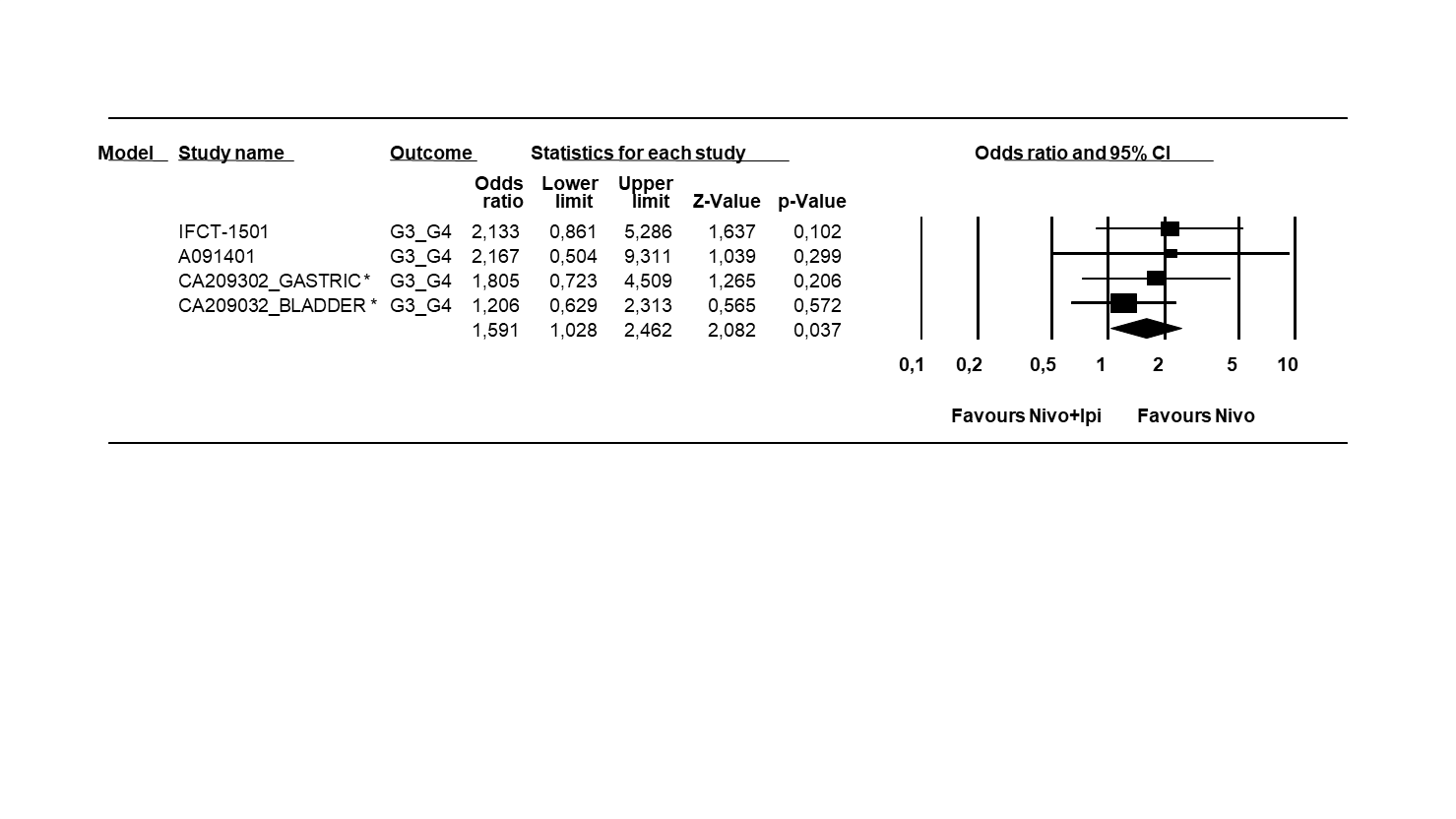


Figure S2 shows G3/G4 toxicity analysis for *NIVO3+IPI1 schedule. Nivolumab resulted less toxic compared to NIVO3+IPI1 combination. Fixed effect model –Heterogeneity not significant (P=0.72). NIVO3/IPI1: nivolumab 3 mg/kg and ipilimumab 1 mg/kg.*
